# Interactive Flexible-Receptor Molecular Docking in Virtual Reality Using DockIT

**DOI:** 10.1021/acs.jcim.2c01274

**Published:** 2022-11-18

**Authors:** Georgios Iakovou, Stephen D. Laycock, Steven Hayward

**Affiliations:** † Digital Platforms, Aviva Plc, Norwich, NorfolkNR1 3NS, United Kingdom; ‡ School of Computing Sciences, University East Anglia, NorwichNR4 7TJ, United Kingdom

## Abstract

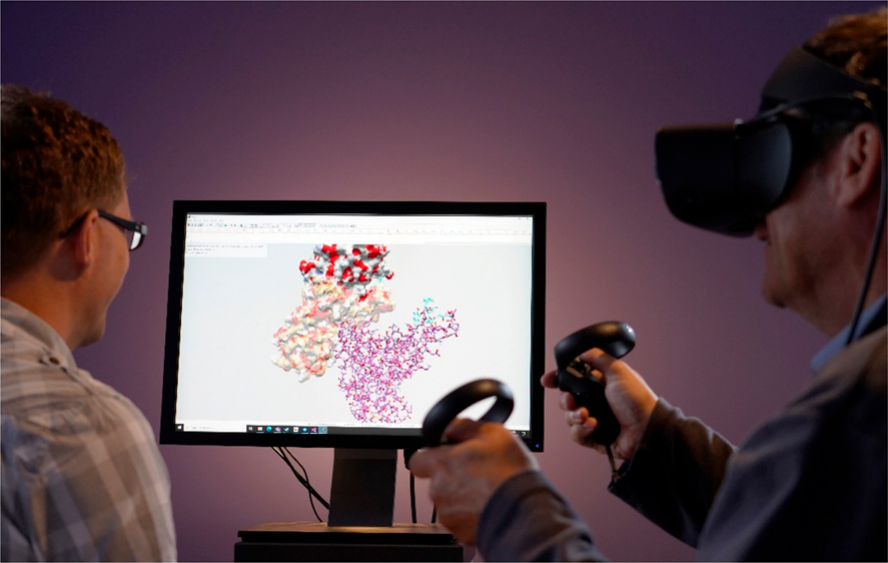

Interactive docking enables the user to guide and control the docking of two biomolecules into a binding pose. It is of particular use when the binding site is known and is thought to be applicable to structure-based drug design (SBDD) and educating students about biomolecular interactions. For SBDD, it enables expertise and intuition to be brought to bear in the drug design process. In education, it can teach students about the most basic level of biomolecular function. Here, we introduce DockIT for virtual reality (VR) that uses a VR headset and hand-held controllers. Using the method of linear response on explicit solvent molecular dynamics simulations, DockIT can model both global and local conformational changes within the receptor due to forces of interaction with the ligand. It has real-time flexible molecular surface rendering and can show the real-time formation and breaking of hydrogen bonds, both between the ligand and receptor and within the receptor itself as it smoothly changes conformation.

## Introduction

Docking refers to the computational process of bringing two molecules together in a binding conformation or pose. Docking can be divided into two categories, automated and interactive. In automated docking, a search is made among the large number of possible binding poses to predict the correct one based on a score derived from a binding energy. A large number of automated docking tools have been developed, popular ones being AutoDock,^1^ Z-Dock,^2^ and HADDOCK.^3^ Autodock is designed for the docking of small molecules to a protein receptor, whereas Z-Dock and HADDOCK can predict protein–protein docking poses. Some of these and other popular automated docking tools have been recently benchmarked against SARS-CoV-2 Protease Mpro.^4^

In interactive docking, a user can manipulate one or more of the molecules using a graphical interface in order to bring them to a binding pose. As it is less likely to be of use for searching among the large number of poses, it is more suited to be used in those cases where the binding site is already known. One such application is structure-based drug design (SBDD) where the binding site of the receptor molecule, usually a protein, is known, and the purpose is to develop a potential drug from a set of lead molecules. In this context, if sufficiently immersive, interactive docking will enable human intuition, expertise, and creativity to be brought to bear. If multiple people can participate or observe the process, it can also foster a collaborative atmosphere within which ideas are nurtured. As almost all biomolecular function involves the association of two or more molecules, interactive docking tools offer an engaging way to teach students about a fundamental process of life.

Several interactive docking tools have been reported in the literature,^5−18^ some of which employ haptic-feedback in order to aid navigation and to sense the force acting on the ligand molecule from the receptor molecule onto which it is being docked so avoiding atomic overlap. A related application uses a haptic device to “dock” atomic models into electron microscopy density maps.^19^

Interactive molecular dynamics (IMD)^20,21^ enables external forces from the user to be applied within a MD simulation, and if these are applied to one of the molecules within a simulation comprising both receptor and ligand molecules in order to bring them into a binding conformation, then it can also be regarded as interactive docking. Naturally, forces can be applied via a haptic device. An advantage of this approach is that the flexibility of molecules is incorporated. The associated disadvantage is that due to the stochastic nature of MD trajectories one cannot exercise direct control over molecular positions and orientations, and if using a haptic device, the forces transmitted to it would fluctuate wildly.

With the advent of more affordable virtual reality (VR) headsets, such as the Quest 2, a fully immersive experience can now be created at relatively low cost compared to previous methods, for example, using a CAVE.^10^ Furthermore, as headsets come with a controller for each hand, one can manipulate the positions and orientations of both molecules in a natural way through hand movements. This makes them particularly suited for docking as the receptor can be attached to one controller with the ligand attached to the other, and it has been shown that tools with this type of VR interface can significantly accelerate the docking process compared to tools that use conventional interfaces.^21^

Here, we report on DockIT^18^ for interactive docking in VR. In addition to the force-based collision detection method, we describe a new space-based collision detection method suitable for rigid docking in the absence of a force field. We also discuss a method to rapidly calculate hydrogen bonds between the receptor and ligand molecules and techniques to rapidly render graphical representations of the molecules, including ball-and-stick, backbone, space-filling, and molecular surface. Its most unique feature is the ability to model receptor flexibility using the method of linear response which in contrast to IMD results in a smooth, time-averaged response.^13^

## Methods

### Force Calculations and Space-Based Collision Detection

Collision detection is a fundamental feature of many 3D simulation software tools that model the physical world as it prevents solid objects from overlapping. We developed two approaches to collision detection. The first approach,^12^ to be used for flexible docking, addresses collision detection and prevents interatomic overlaps using the magnitude of the interaction force and a threshold value which when exceeded reports a collision. The interaction energy and force are computed on the GPU using the method described in Iakovou et al.^22^ (developed for force rendering on a haptic device) and accounted for nonbonded interactions within a cutoff distance of 8 Å. In contrast to previous methods that use a force grid, the method calculates the intermolecular forces in real time and is therefore suitable for the treatment of flexible molecules. The van der Waals (vdW) and electrostatic interactions are modeled using the 12-6 Lennard-Jones (LJ) potential and Coulomb’s law, respectively, with the LJ parameters and charges being obtained from the Gromos54a7 or Amber03 force fields selected using the pdb2gmx command of the MD simulation package, GROMACS.^23^ To compute the interaction energy and force, the method constructs a regular grid for the ligand on the CPU and copies this structure as a 1D array to the GPU along with other information such as the list of receptor atoms, a transformation matrix for the receptor atoms, a user-defined cutoff distance, nonbonded force parameters, etc. According to Iakovou et al.,^22^ a regular grid-based approach performs better than the octree-based approach on the GPU. Using this information, the method queries the regular grid in parallel for each receptor atom, identifies those ligand atoms within the cutoff distance, and computes the force on each receptor atom from the ligand. The force on each receptor atom is needed to calculate the atomic displacements for the flexible receptor. To calculate the total force on the receptor from the ligand or vice versa, the force on each receptor atom is calculated and then summed in groups of 256 atoms on the GPU, and these group subtotals are transferred to the CPU and summed. The method can perform force calculations in less than 2 ms for very large molecules (comprising hundreds of thousands of atoms each), and it can be used for handling collision detection during interactive docking simulations in VR. Note that DockIT uses a distance-dependent relative permittivity^24^ in order to model the screening effect of the water solvent on electrostatic interactions.

For rigid docking the method’s dependency on the pdb2gmx tool is a limiting factor that prohibits users from running interactive rigid docking directly on Protein Data Bank (PDB) files. To remove this limitation, we developed a second collision detection approach that detects spatial overlaps based on vdW radii of the atoms. This follows the same execution steps used by the force-based one with the only exception that instead of computing the total interaction force it computes and returns the maximum interatomic penetration distance measured between a receptor atom and those ligand atoms within the cutoff. However, as mentioned, when using a flexible receptor, force-based collision detection should be used, and this is set as the default.

### Modeling Receptor Flexibility Using Linear Response

Receptor flexibility is modeled using the method of linear response as described previously.^13^ Linear response states that the equilibrium fluctuations of the unperturbed system (receptor without ligand) can be used to approximate the response of the system under external forces, e.g., forces from a ligand. Although there are several ways to model the fluctuations, we opted for an accurate method: MD simulation of the receptor in an explicit solvent. In evaluating atomic displacements on the receptor atoms due to the external forces from the ligand, it was shown that by performing an eigenvector decomposition of the variance–covariance matrix of atomic fluctuations, and by using only the first M eigenvalues and eigenvectors, memory and interactive time limits could be met by reducing M. This exploits the concept of the important subspace in protein dynamics, whereby most of the fluctuation occurs within a relatively small subspace, i.e., for small M. For example, for maltose binding protein (MBP), previously we found that although we could only use 3.2% of the total number of eigenvectors and eigenvalues, this accounted for 87% of the total fluctuation. In static equilibrium,
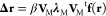
1where, **r** = **r**_**o**_ + **Δr**. Equation 1 gives the atomic displacements, **Δr** (3N × 1 matrix), from the relaxed structure, **r**_**o**_ (3N × 1), to the deformed structure, **r** (3N × 1), due to the forces, **f**(**r**) (3N × 1) on the deformed structure. **λ**_M_ (M × M) is the diagonal matrix of the first M eigenvalues, and **V**_M_ (3N × M) is the matrix of corresponding eigenvectors (the superscript t denotes the transpose). β is 1/*k*_b_T, where *k*_b_ is Boltzmann’s constant and T the absolute temperature. This equation means that the external forces on the receptor from the ligand are balanced by the restoring forces within the deformed receptor. An iterative procedure is used to move smoothly toward this state.^13^ To perform flexible docking, the user needs to provide the eigenvalue and eigenvector files in a specified format. One also needs to supply the coordinates of the relaxed structure, , loaded as the “Receptor” at the start of a DockIT session, here the “closest to average” structure from the MD trajectory. Further details are provided in the article describing the Haptimol FlexiDock prototype^13^ and the user manual.

### Real-Time Visualization of the Molecular Surface

Visualizing the three-dimensional shape of molecular structures and being able to identify features within them is important for interactive molecular docking. We employ four main graphical depictions: space-filling, ball-and-stick, backbone, and the molecular surface. Rendering the molecular surface is very useful for indicating potential binding sites as these can appear as cavities. To improve efficiency, we employ a deferred-rendering approach, and for the depictions (excluding the molecular surface), we use billboards for each cylinder and sphere.

Our approach for rendering the molecular surface or more explicitly, the solvent excluded surface (SES), is based on a GPU-accelerated version of the work by Kim et al.^25^ The Marching Cubes algorithm is used to compute a triangle mesh for the SES using cubes with side lengths of either 0.375 or 0.75 Å depending on the GPU memory and size of the structure. This method is sufficiently fast for rendering the flexible molecular surface of the receptor in real time.

### Controlling Docking Simulations in VR

**Figure 1:**
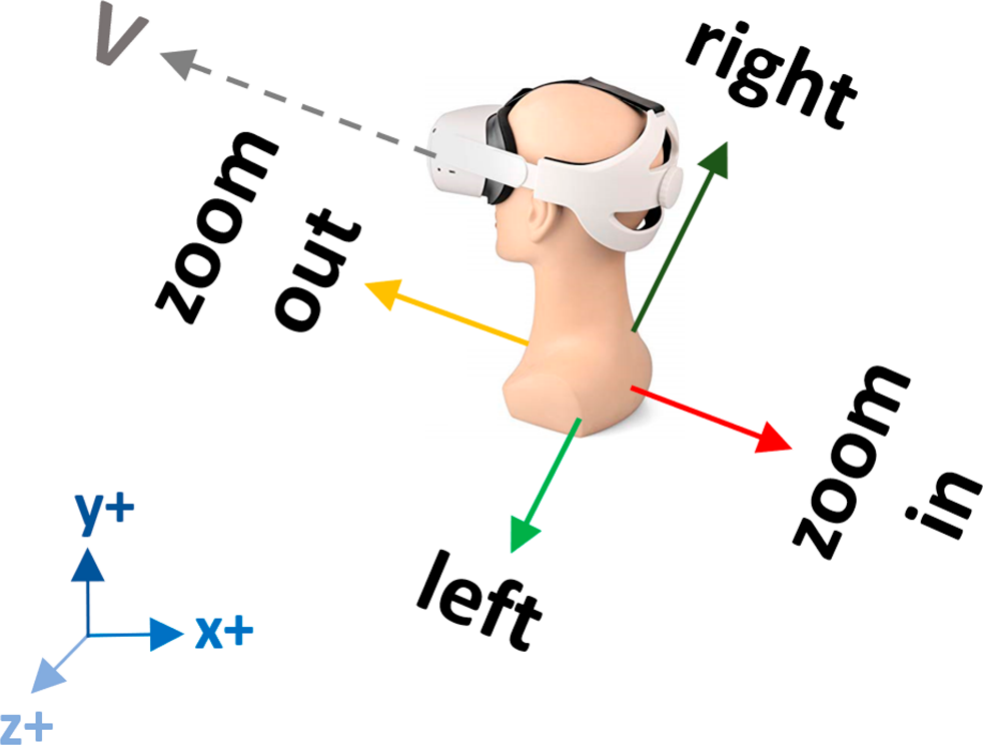
Depicting how global left/right and zoom in/out movements are applied relative to the viewing direction **V** of the headset. Even though the left/right and zoom in/out displacements received from the controllers are along the X and Z axes shown at the bottom-left corner (i.e., scene’s world coordinates), we transform those displacements relative to the viewing direction vector **V**, using the headset’s orientation matrix, and then apply the new transformed displacements (i.e., vector components along the X and Z axes) to the scene.

**Figure 2:**
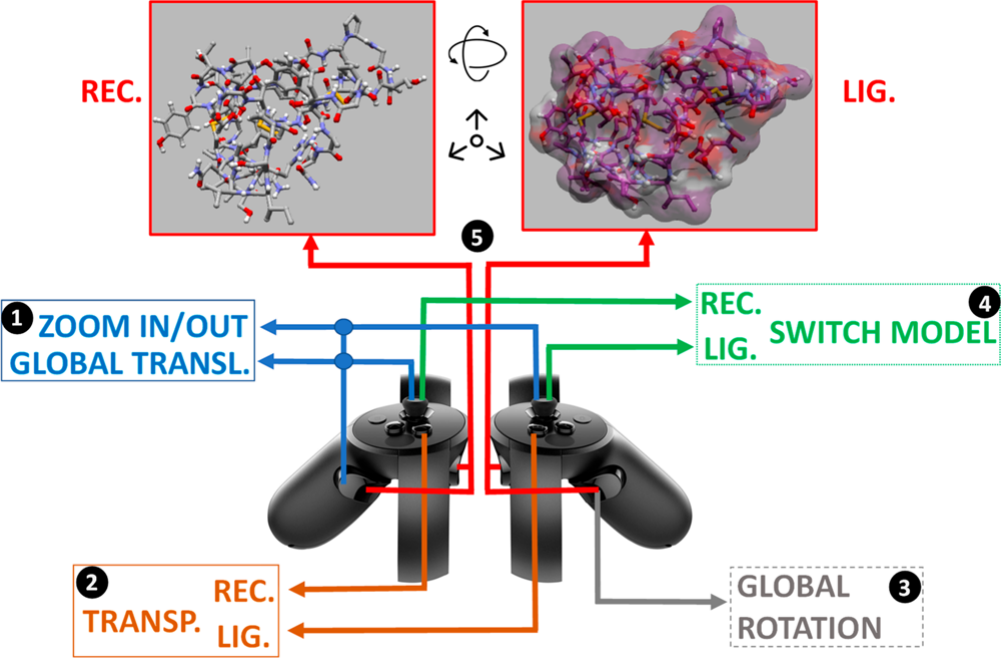
Oculus Touch controllers and buttons used for navigating a VR-based interactive docking simulation in DockIT. (1) Left hand-grip + thumb-stick and/or left hand-grip + right thumb-stick translate the scene to “globally” left/right/up/down and in/out, respectively. (2) Y and B buttons enable/disable surface transparency for the receptor and ligand, respectively. (3) Right hand-grip while rotating the right controller rotates the scene “globally”. (4) Pressing left thumb-stick and/or right thumb-stick switches the molecular representation of the receptor and ligand, respectively. (5) Left trigger + left hand-grip moves and rotates the receptor, whereas the right trigger + right hand-grip moves and rotates the ligand.

We implemented support for the Oculus Touch controllers. These are affordable consumer-level dual controllers (one for the left hand and the other for the right hand), that act as colocated virtual hands offering 6DOF control. We find these devices very suitable for VR-based interactive docking simulations since they provide an intuitive way to move, rotate, and interact with the molecules, enhancing the overall user experience. In our approach, we attach each controller to one of the molecules (left for receptor and right for ligand) at the molecule’s center of mass. Each controller allows the user to change the 3D molecular representation (e.g., space-filling, ball-and-stick, etc.) of the respective molecule by clicking the thumb-stick. Surface transparency can also be toggled on/off by pressing the Y button for the receptor and the B button for the ligand. To move and rotate a molecule in 3D space, the user must press the controller’s trigger and hand-grip buttons, causing the controller’s positional and rotational changes (from that point on) to be applied to the molecule’s transformation matrix. When the two molecules collide, the application stops updating the molecules’ transformation matrices similar to the method described in Iakovou et al.,^12^ and both controllers vibrate using the non-buffered haptics approach described in the Oculus Touch documentation. This warns the user to stop attempting to overcome molecular repulsion. By releasing the trigger and hand-grip buttons, repositioning the controller in space, and pressing the same buttons again, the user can apply a series of successive movements on each molecule and displace it large distances within the virtual world. The controllers can be used to apply a “global” translation and rotation to both molecules, enabling the user to inspect the interacting molecules from various angles, depths, and heights. By pressing the grip button on the left controller and moving the left thumb-stick left/right and up/down, the user can translate both molecules along the X and Y axes, respectively. If the up/down movement is applied on the right thumb-stick, the user can zoom in on and zoom out from both molecules along the Z axis, with all axes being relative to the viewing direction of the headset (Figure 1). We compute and apply this “global” X, Y, and Z movement to allow the user to rotate the headset by 360° without compromising the direction of the “global” displacement exercised by the controllers. For example, if the X, Y, and Z movement was not relative to the headset’s viewing direction and the user rotated the headset by 180° (during a virtual session), then all “global” displacements along the X and Z axes would be inverted, causing the molecules to move in opposite directions to expected. Lastly, “global” rotation (using the receptor as the center of rotation) can be applied to both molecules by pressing the right-hand trigger button while rotating the controller. Apart from the initial calibration step required by the Oculus setup, no additional or periodic calibration of the Touch controllers is necessary. Figure 2 illustrates how the Oculus Touch controllers can be used in DockIT for control and navigation.

### Real-Time Calculation of Hydrogen Bonds

Visualizing hydrogen bonds between the receptor and ligand as they form during an interactive docking simulation is important as their indication can help identify the native binding pose and can provide valuable visual cues for the study and understanding of molecular docking to students. We opted to use criteria derived by McDonald and Thornton^26^ based on an analysis of a large number of high-resolution X-ray structures of proteins. These criteria identify a primary hydrogen bond when the distance between the hydrogen and the acceptor atom is less than 2.5 Å and the angle between the line from the donor atom to the hydrogen and the line from the hydrogen atom to acceptor atom is greater than 90°.

To satisfy the high frame rates required for rendering on a standard HMD device, we have developed a GPU-accelerated method that can achieve those rates and computes the formation of hydrogen bonds for very large structures comprising hundreds of thousands of atoms. The method utilizes the hydrogen atoms that may be already present in the PDB file or placed by the GROMACS pdb2gmx tool.^23^ Using a predefined map of donor and acceptor atom names, the method flags the donor and acceptor atoms during PDB-file loading, creates the donor and acceptor atom lists, and copies this information to the GPU. The method traverses in parallel the list of acceptor atoms for each donor atom and identifies all acceptor atoms within a 2.5 Å distance and a donor–hydrogen–acceptor angle greater than 90°, returning a list of donor–acceptor atoms pairs to the CPU for visualization.

### Implementation

DockIT^18^ is a windows-based application implemented with Visual C++, utilizing the Windows Standard Development Kit library (win32 SDK) for its graphical user interface, OpenGL for the rendering of the 3D molecular models, and the OpenCL library for programming the GPU. The application supports the Oculus Rift, Oculus Rift S, and Meta Quest2 (with link cable) HMD devices and the Oculus Touch Controllers, which are integrated using the Oculus Native PC SDK library. Support for the Geomagic Touch haptic device is also provided and implemented using the OpenHaptics toolkit from Geomagic (not available in VR mode).

### Additional Features

In addition to the those described above, DockIT has other useful features. One can switch on and off the three individual components to the force: the vdW repulsive, the vdW attractive, and the electrostatic. Another useful feature enables the user to monitor the distance between selected pairs of atoms, when, for example, they have been determined by experimental methods such as NMR, FRET, EPR, or cross-linking studies. It has a “ghost” facility which allows one to see but not collide with, or feel using haptics, selected regions. This can be useful when one cannot access a binding pose due to blocking regions. One can also monitor live the total interaction energy between the receptor and ligand which is presented in a graphical format. A useful feature is the ability to record the paths of the receptor and ligand during a docking session. The replay of a docking session viewed from different directions and positions in VR, and using different molecular models, can give a whole new perspective. The application can load files either in PDB or mmCIF file formats but can save only in the PDB format. This saving capability coupled with the ability to combine and treat a docked receptor–ligand complex as a new “receptor” while allowing the user to load a new ligand (without requiring the user to close the application) makes it a practical tool for rapidly building large multicomponent systems that could be used for MD simulations or other purposes. During a VR session, the user has the option of utilizing DockIT’s user interface in 2D using the Oculus Dash or a subset as fully integrated VR menus.

## Results and Discussion

The results of testing on three different GPUs are included here to show the performance of a test case. We recorded a simulation of MBP (comprising 5737 atoms) in interaction with maltose (comprising 45 atoms). Using the VR controller, we moved maltose in and out of the potential binding site observing the conformational change. Each test involved playing back the same simulation in both VR mode and in standard 2D mode and recording the frame rate every second. The average frame rates are shown in Table 1. The frame rates include the collision detection, hydrogen bond calculations, force calculation, receptor conformational response, and cost of rendering either the surface or ball-and-stick model. In the case of rendering a flexible molecular surface for the receptor, it must first be recalculated based on the position of the atoms in the receptor making it the most costly graphics representation.

**Table 1:** Comparison of Frame Rates for Interactive Docking Simulations of MBP (5373 atoms) and Maltose (45 atoms) on Different Computers^s^

	Average frame rate with molecular surface (fps). Average fps in VR mode included in brackets	Average frame rate with ball-and-stick rendering (fps). Average fps in VR mode included in brackets
NVidia GeForce GTX980 andIntel i7-10700 CPU @ 2.90 GHz	90.18 (54.38)	414.44 (80.97)
NVidia GeForce RTX2080 andIntel i7-10700K CPU @ 3.8 GHz	152.58 (81.00)	539.67 (81.08)
NVidia Quadro P5000 andIntel i7-7700HQ CPU @ 2.8 GHz (laptop)	72.37 (40.95)	263.67 (80.95)

s The frame rate includes collision detection, hydrogen bond calculations, force calculation, receptor conformational response, and cost of rendering the molecules in either surface or ball-and-stick model.

### Docking Experiments

Four tutorials are provided with the installation, three for flexible receptor docking and one for rigid docking. The flexible docking tutorials will be useful for teaching students about the crucial role conformational change plays in biomolecular function. The tutorials use all the features described above and give the user experience of the capabilities of DockIT.

#### Tutorial: “Docking Maltose to MBP”

**Figure 3:**
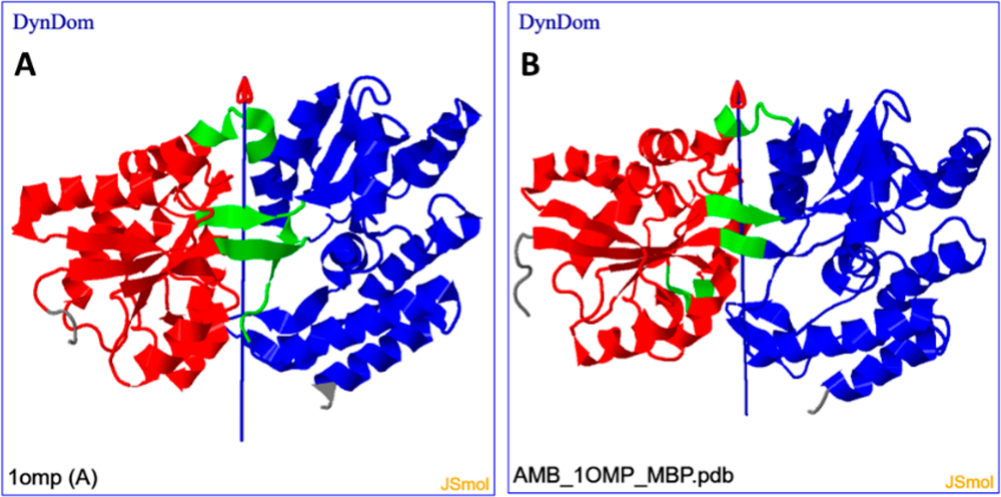
(A) DynDom result for movement between maltose-free (PDB: 1OMP) and maltose-bound (PDB: 1ANF) structures of MBP, indicating domains (blue and red), hinge-bending regions (green), and hinge axis. (B) DynDom result for movement between relaxed and maltose-docked structures.

This tutorial concerns the docking of maltose to MBP. The response matrices in eq 1 were determined from a 100 ns explicit solvent MD trajectory of maltose-free MBP (PDB: 1OMP). The tutorial uses 26 eigenvalues and eigenvectors (M = 26). To perform docking, distances between selected pairs of atoms from maltose and MBP, as determined from the maltose-bound structure, were monitored. The aim is to bring these as close to their values in the bound structure as possible. In maneuvering maltose into the cleft in MBP, a domain movement occurs. It is instructive to compare this domain movement to the domain movement between the maltose-free and maltose-bound crystallographic structures. To make this comparison, the DynDom program^27^ was used at the DynDom web server.^28^ DynDom assigns domains, hinge-bending regions, and hinge axes based on the conformational change between two structures. As seen in Figure 3, there is a remarkably good correspondence between docking and experimental results in the assignment of domains and hinge-bending regions, as well as the hinge axis location and orientation. There is, however, a difference in the hinge-bending angle of rotation, as docking results in a 22° rotation angle compared to 36° in the experimental case.

#### Tutorial: “Docking Glutamine to GBP”

This tutorial concerns the binding of glutamine to glutamine binding protein (GBP). The response matrices were determined from a 100 ns explicit solvent MD trajectory of glutamine-free GBP (PDB: 1GGG). The tutorial uses 100 eigenvalues and eigenvectors (M = 100). A similar result to the binding of maltose to MBP was found.

#### Tutorial: “Dynamic Salt Bridge Formation and the Electrostatic Interaction”

This tutorial is suitable for teaching students about the electrostatic nature of salt bridges in proteins and the ability of molecules to change conformation in forming them. It illustrates the use of the facility to switch on and off any of the three components of the nonbonded interaction, in this case the electrostatic component. In the relaxed state of GBP, Lys214 has a salt bridge with the side chain of Glu211 indicated as a hydrogen bond in DockIT. Bringing the glutamine ligand close to Lys214 causes Lys214 to move toward the ligand to form a salt bridge with its C-terminal carboxyl group, breaking its bridge with Glu211 in the process. Switching electrostatic interactions off breaks this bridge, and Lys214 relaxes back to its original position reforming its bridge with Glu211. Switching the electrostatic interactions back on causes Lys214 to move back to form the bridge with the ligand.

#### Tutorial “Docking of an Antibody to SARS-CoV-2 Spike Protein”

This tutorial concerns the rigid docking of an antibody to the receptor-binding domain of the SARS-CoV-2 viral spike protein. It requires extensive use of the ghost feature as without it docking cannot be achieved. It illustrates how one can use this feature to find regions that must undergo conformational change upon binding.^29^

### User Survey Comparing Standard Input of Keyboard and Screen with VR

We conducted a small user study in three different research groups involving 12 people. Most were postgraduates, studying or researching computational structural biology; the remaining were in other disciplines of the computing sciences. One third had prior experience in molecular docking, and one-quarter had experience in interactive molecular docking. We asked all participants to attempt to dock maltose to MBP using the standard input of a keyboard with a mouse and compare it to performing the same task in VR. In response to the question “Navigation using DockIT in VR is easier than navigation with a keyboard and mouse”, four strongly agreed, five agreed, two were neutral, and one thought the keyboard and mouse was better. When asked about the advantages, several commented on the more intuitive nature of VR interaction for controlling the molecules, a finding that is in accordance with a previous study.^21^ Disadvantages of VR included initial difficulty in understanding the controls and the precision of the mapping between hand movements and molecular movements.

## Conclusions

The DockIT tool for interactive docking of two molecules in VR has been presented. We have described the underlying methods that enable the tool to be used for rigid and flexible-receptor docking. These methods are designed and implemented in ways that exploit features of the modern GPU to achieve maximum efficiency both in memory and computation time. The methods include real-time evaluation of forces on the receptor atoms from the ligand, real-time evaluation of the conformational change of the receptor due to these forces, real-time rendering of the molecular surface due to this conformational change, and real-time evaluation and depiction of hydrogen bonds as they form and break. The iterative approach taken to reach static equilibrium produces a pleasingly smooth response which stands in contrast to the IMD approach. However, the linear response approach does not prevent occasional unrealistic distortions in the bonded structure.

The benefit of performing docking within VR is that it mimics what one would naturally do when trying to fit two objects together in the real world which humans are naturally good at. Using the touch controllers naturally overcomes the colocation problem for which there is no easy solution when using a mouse and keyboard or a haptic device. Our user study indicates the benefit of using VR over a standard keyboard and mouse for interactive docking.

Interactive docking will be of use for those cases where the binding site is already known. Applications for flexible receptor docking with DockIT could be in SBDD where response matrices from a single MD simulation can be used to test the docking of multiple candidate drug molecules. As the ligand is currently modeled as rigid, it may be particularly suited to fragment-based drug design where fragments are often rigid. An obvious application is in education where in VR it can teach students in an engaging way about molecular interactions, the forces that govern them, and the shape changes biomolecules undergo upon binding.

## Data and Software Availability

Software Download: DockIT is available at http://www.haptimol.co.uk/downloads.htm.

## Supporting Information Available

Video of DockIT being used in VR for flexible docking (MP4)
